# Using wire shaping techniques and holographic optics to optimize deposition characteristics in wire-based laser cladding

**DOI:** 10.1098/rspa.2016.0603

**Published:** 2016-12

**Authors:** N. J. Goffin, R. L. Higginson, J. R. Tyrer

**Affiliations:** 1 Wolfson School of Mechanical, Electrical and Manufacturing Engineering, Loughborough University, Loughborough, LE11 3TU, UK; 2 Department of Materials, Loughborough University, Loughborough, LE11 3TU, UK

**Keywords:** laser cladding, wire shaping, power density, dilution, holographic optics

## Abstract

In laser cladding, the potential benefits of wire feeding are considerable. Typical problems with the use of powder, such as gas entrapment, sub-100% material density and low deposition rate are all avoided with the use of wire. However, the use of a powder-based source material is the industry standard, with wire-based deposition generally regarded as an academic curiosity. This is because, although wire-based methods have been shown to be capable of superior quality results, the wire-based process is more difficult to control. In this work, the potential for wire shaping techniques, combined with existing holographic optical element knowledge, is investigated in order to further improve the processing characteristics. Experiments with pre-placed wire showed the ability of shaped wire to provide uniformity of wire melting compared with standard round wire, giving reduced power density requirements and superior control of clad track dilution. When feeding with flat wire, the resulting clad tracks showed a greater level of quality consistency and became less sensitive to alterations in processing conditions. In addition, a 22% increase in deposition rate was achieved. Stacking of multiple layers demonstrated the ability to create fully dense, three-dimensional structures, with directional metallurgical grain growth and uniform chemical structure.

## 1. Introduction

Laser cladding is the term used to describe the use of a laser to coat the surface of one material with a different material, in order to alter the surface properties. This has a variety of applications, such as increasing corrosion resistance and/or hard facing, with other possibilities such as component repair and manufacturing.

The majority of commercial efforts have been focused on powder-based laser cladding, with powder-fed systems such as LENS™ and direct metal deposition (DMD) [1]; however, some research has been conducted into the use of wire as a source material. Experiments by Kim *et al.* [2] showed the importance of the feed angle of the wire and its direction relative to the cladding direction. Their work was corroborated by Mok *et al.* [3], whose experiments with wire feeding found a preferred feed angle of 45–60°. A further parametric study by Abioye *et al.* [4] on Inconel wire deposition showed that the contact angle between the wire and substrate was not only a function of feed angle but also of wire feed rate and substrate traverse speed and could be modified by those parameters, independently of the feed angle setting. Important observances were made by Miranda *et al.* [5] regarding the relative diameters of beam and wire. Using a laser beam similar in width to the wire is more productive, because less heat is lost to the substrate. However, a larger beam increases the melting area and improves the stability of the process with regard to process fluctuations. Kim & Peng [6] showed that dilution can be controlled by reducing the input energy over the course of the cladding process to compensate for heat build-up.

### (a) Comparisons between wire and powder-fed deposition

Wire feeding, in general, is not highly regarded in laser-based cladding. For example, the book by Toyserkani [7] devotes only a single short subsection to wire deposition, while the rest of the book is devoted to powder feeding. However, it is a standard procedure in arc-based cladding, with a number of turn-key wire-based tungsten inert gas (TIG) deposition systems commercially available. Williams *et al.* [8] demonstrated the ability to use a metal inert gas (MIG)-based system to create high-quality large components, including unsupported overhanging structures. This process is a ‘near-net shape’ manufacturing process, requiring post machining for a completed structure. Wire feeding with lasers appears to be regarded as an interesting curiosity rather than a viable process, despite its ubiquity in industrial TIG and MIG deposition.

The primary reason for the focus on powder-based deposition with lasers appears to be that it is physically easier to achieve high-quality results with a powder system. A number of researchers have noted several major disadvantages with wire compared with powder feeding. It is highly sensitive to process parameters [7], and flaws such as porosity, cracking and high dilution are much more common with wire than with powder [9]. Others, however, have noted that wire also possesses major advantages as a source material: wire feeding has a much greater deposition efficiency; close to 100% of the delivered material is deposited, as opposed to around 40% for powder [10]. Wire feeding is more suited to automated production, being more easily regulated than powder and a wire feeder is more easily adapted to awkward cladding positions, such as the insides of tubes [2] or vertical or overhead cladding positions. The overall principle is that while using wire gives a superior deposition process to powder, high-quality results are harder to achieve. As a result, laser wire deposition has somewhat fallen by the wayside.

### (b) Use of shaped wire

The use of wire shaping techniques to alter the cross-sectional shape of the wire has received little experimental attention thus far, but has demonstrated potential to alter this paradigm. Rolls Royce experimented with this technique in order to maximize heat absorption, as detailed in their patent EP 1454703 B1 [11]. This proposes the use of preheated rollers to alter the cross-sectional shape of the wire just prior to processing, easing the reshaping of the wire and reducing the work hardening effect. Other research by Fux & Luft [12] investigated the creation and use of amorphous metal ribbons for laser deposition, using a rapidly scanned laser to cover the whole width of the ribbon. This was not wire in the traditional sense but was successful in creating low-dilution clad tracks.

In this work, experimental studies of laser deposition using shaped wire are presented. Holographic optical elements (HOEs) [13] have previously been used to control the laser beam thermal distribution in laser welding [14], powder-bed laser cladding [15] and laser cladding with pre-placed circular section wire [16], showing the ability to control the shape and fluid flow within a melt pool. Building on previous wire work, the use of wire shaping seeks to further develop the process by optimizing the wire shape to give uniform heat transfer from wire to substrate across the width of the heat affected zone, HOEs were used to give a uniform laser profile. Experimentation is presented in two phases:

— The first phase picks up where Goffin *et al.* [16] left off, using pre-placed wire for the purposes of directly comparing heat flow between the two wire shapes in order to study the heat flow and track formation on their own, in isolation from realistic processing conditions.
— The second phase incorporates a wire feeder into the process and compares the two wire shapes under realistic processing conditions without using HOEs. These results are then developed with the demonstration and characterization of multilayer stacking using shaped wire.

## 2. Material and methods

For the laser cladding experiments, two types of mild steel substrate were used: 0.8 mm thick for pre-placed experiments and 2 mm thick for wire-fed experiments. Material compositions are given in tables 1 and 2 respectively.

**Table 1.** Alloy composition of 0.8 mm thick mild steel (weight %).

element	Mn	C	Cr	Cu	S	P	Ni	Fe
% weight	0.791	0.154	0.079	0.045	0.018	0.017	0.017	balance

**Table 2.** Alloy composition of 2 mm thick mild steel (weight %).

element	Mn	C	P	S	Si	Al	Fe
% weight	0.359	0.054	0.014	0.012	0.006	0.0058	balance

The substrate plates were cut into individual coupons to allow multiple depositions per plate, while eliminating heat conduction between them, in a similar way to previous work by Higginson *et al.* [15].

**Figure 1.**
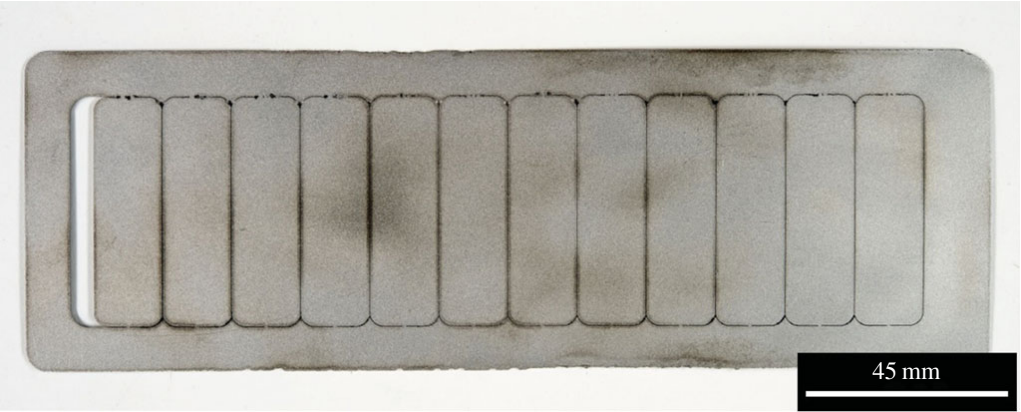
A 0.8 mm thick substrate with 12 individual sample coupons cut out.

**Figure 2.**
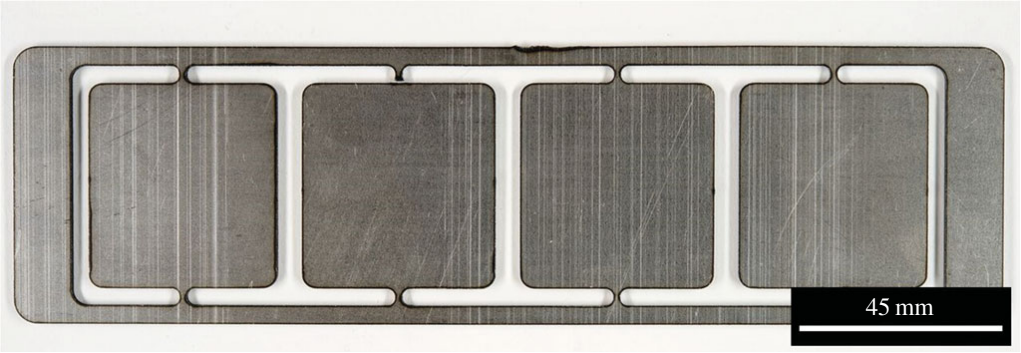
A 2 mm thick substrate with individual sample coupons cut out.

Figures 1 and 2 show the substrate sample plates with coupons cut out. Each coupon was attached by 1 mm wide tabs at each end. The plate in figure 1 was designed for pre-placed single tracks and therefore the coupons were long and narrow. When wire feeding was implemented, substrates were altered to the design shown in figure 2. This was to allow multiple tracks to be created on a single coupon, which was used to evaluate the relative sensitivity to processing conditions of the two wire shapes, by placing tracks in different places on the substrate.

Two forms of 316 stainless steel wire were used for experiments. In both cases, the alloy composition was the same and is given in table 3.

**Table 3.** Alloy composition of AISI 316 stainless steel (weight %).

element	Cr	Ni	Mo	Mn	Si	Cu	P	C	S	Fe
% weight	18.26	12.09	2.53	1.61	0.42	0.12	0.02	0.11	0.01	balance

**Figure 3.**
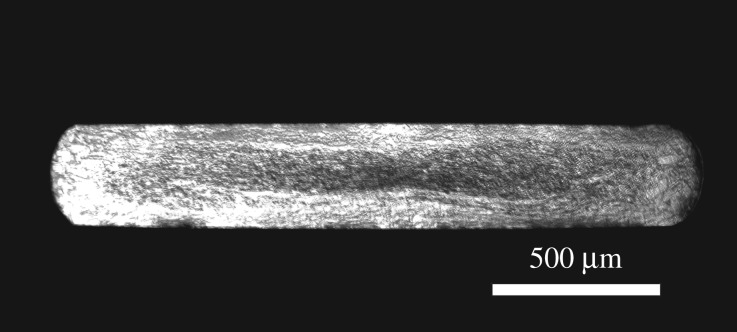
Optical micrograph of shaped wire cross-section etched with Kalling's No.2 reagent.

Standard 1 mm diameter wire was used in both pre-placed and fed wire experiments. This was then converted into flat wire with dimensions of 2 mm wide by 0.3 mm thick. A sectional micrograph is given in figure 3.

To create the flattened wire, the procedure below was followed:

— The wire was annealed in a box furnace at 900°C for 1 h, to relieve residual stresses left over from the original manufacturing and coiling of the wire.
— The annealing process resulted in a thick coating of oxide on the surface of the wire. This was removed by immersion in an 11% solution of sulfuric acid held at 58°C for 30 min and then mechanically cleaned in an ultrasonic bath.
— The annealed wire was passed through a Durston D2-130 manual rolling mill, to create a flattened cross-section.

The flattening process also resulted in a reduction in the cross-sectional area of the wire and thus the volume per unit length. Flat wire clads were therefore physically smaller than their round wire counterparts:

— round wire: 0.78 mm^2^ of material per millimetre of wire length;
— flat wire: 0.6 mm^2^ of material per millimetre of wire length.

To take account of this, the clad tracks were analysed using relative properties such as dilution and profile, rather than absolute dimensions such as width or height.

### (a) Laser equipment

**Figure 4.**
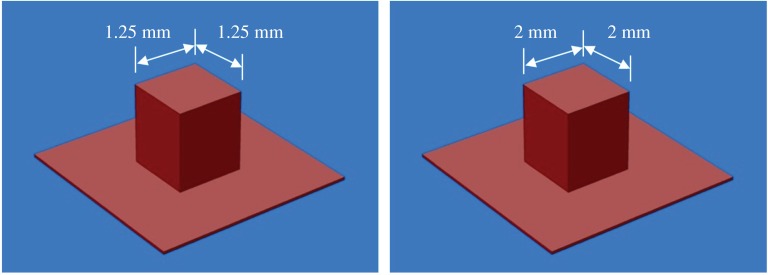
Plots of HOE beam shapes used for CO_2_ laser experiments.

For deposition experiments, two types of laser were used. Pre-placed wire experiments used a Coherent Everlase S48 CO_2_ laser rated at 1.2 kW. This was equipped with the same HOE optical system used by Goffin *et al.* [16]. HOEs were used to create pedestal beams, which were used for both wire types: a 1.25 mm square beam for 1 mm diameter round wire and a 2 mm square beam for shaped wire. Plots of these are shown in figure 4. The pedestal beam is a simple profile that eliminates the two primary weaknesses of traditional focused TEM_00_ beams, namely their circular spot and non-uniform energy profile. Further explanation of this can be found in the aforementioned publication. Cladding was carried out using a continuous wave (CW) beam.

**Figure 5.**
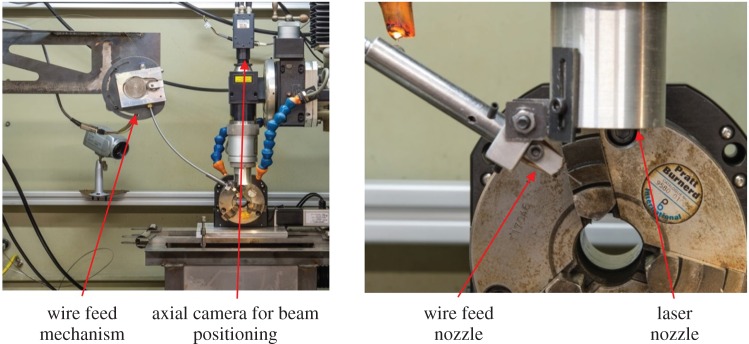
Images showing the wire feeding mounted on the laser system.

For wire feeding experiments, it was necessary to use an Nd:YAG laser which was not capable of mounting holographic optics. For this, a focussing lens with 120 mm focal length was used and the beam was expanded to 4 mm diameter. This was considered to be a suitable substitute for a pedestal beam, in line with results for expanded beams found by Goffin *et al.* [16]. The Nd:YAG laser, with wire feeder attached is shown in figure 5.

Certain features of the Nd:YAG system made it more suitable for wire feeding. The system was integrated with a higher precision CNC system than the CO_2_ laser and was also fitted with an axially mounted camera equipped with a cross-hair and TV system, giving extremely precise beam position.

For Nd:YAG laser cladding, arrays of single tracks were created for both wire types and the results compared. The settings used are given in table 4.

**Table 4.** Nd:YAG single layer clad track settings for round and flat wire feeding.

	1 mm diameter round wire	1.9 × 0.3 mm flat wire
start delay (s)	20	20
traverse rate (mm min^−1^)	55	55
beam diameter (mm)	3.5	3.5
frequency (Hz)	75	75
pulse length (ms)	6	6
pulse energy (J)	3.63	3.63
average power (W)	274	274
wire feed delay (s)	17	18
wire feed rate (mm s^−1^)	1.85	3.10

### (b) Wire feed implementation

The wire feeder design utilized a custom designed feeding mechanism driven by a stepper motor and controlled using a custom designed user control interface. This allowed the selection of the wire feed rate, starting delay time and total feed length and feed time; along with the ability to govern the ramp up and ramp down rates and the ability to retract the wire a short distance once deposition was complete. The control system also allowed for manual feed and retraction so as to accurately adjust the wire position.

For round section wire, a standard MIG welding extrusion nozzle was used. For the feeding of shaped wire, a separate nozzle was modified with a slotted mask to hold the wire in position.

### (c) Characterization techniques

The primary method for characterization was optical microscopy, with a small amount of scanning electron microscopy (SEM) also used. In order to prepare the samples for characterization, they were sectioned, mounted in Bakelite, ground on a series of progressively finer silicon carbide papers and then polished to a surface finish of 1 µm using a diamond suspension solution.

Optical micrographs were taken using a Nikon Optiphot microscope with a QImaging Micropublisher camera, controlled with Image-Pro Premier 9.1 imaging software. Analysis utilized bright field (BF), dark field (DF) and diffractive interference contrast (DIC) illumination techniques in order to extract different types of data from the samples. Three etchants were used: Kalling's No.2 reagent for general microscopy, Schaftmeister's reagent [17] for stainless steel grain boundaries and 2% Nital for mild steel grain boundaries.

The SEM was used in order to conduct electron back-scatter diffraction (EBSD) and energy-dispersive X-ray spectroscopy (EDS). EBSD required a far greater level of sample surface quality. For this, colloidal silica was used to polish the sample to a surface finish of 0.02 µm. After this, they were rinsed and cleaned with methanol with final cleaning completed using an ozone cleaner.

EBSD uses back-scattered electrons from the surface of the sample to create sample maps that measure crystallographic grains. For this study, two types of maps were used: inverse pole figure (IPF) maps used to measure grain sizes and orientations, and phase maps used to distinguish between the face centred cubic (FCC) austenitic stainless steel structure and the body centred cubic (BCC) ferritic mild steel structure. These maps were created using a LEO 1530VP Gemini FEG-SEM fitted with a NordlysNano EBSD detector and AZTec software. The AZTec software was capable of simultaneous EBSD and EDS analysis, and this process was used for these investigations. EDS phase maps were created for the major alloy constituents, Fe, Cr and Ni, as well as for O, in order to evaluate the effects of oxide formation.

### (d) Quantitative clad track characterization

Evaluations of wetting and measurements of dilution were both plotted with applied power density in order to compare the experimental results for pre-placed cladding. The definition of dilution used was identical to that defined in Goffin *et al.* [16].

## 3. Pre-placed experimental comparison between round and flat wire

**Figure 6.**
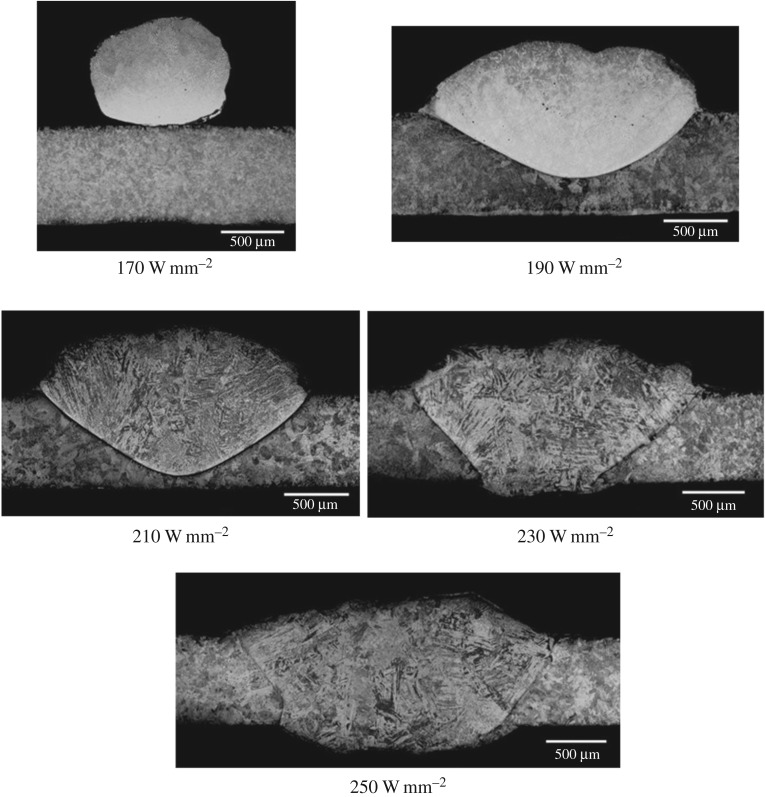
BF optical micrographs of clad tracks created with a 1.25** **mm square pedestal beam on pre-placed 1** **mm round wire at increasing power densities etched with Kalling's No.2 reagent. Reproduced from Goffin *et al*. [16].

Figure 6 shows clad tracks created with pre-placed wire and a pedestal laser beam at a range of increasing power densities. Below 170 W mm^−2^, heating was not sufficient to adhere the wire to the substrate. Above 250 W mm^−2^, dilution was high enough that the track could no longer be considered as a separate material from the substrate. These two power density levels therefore represented the limits of experimentation.

**Figure 7.**
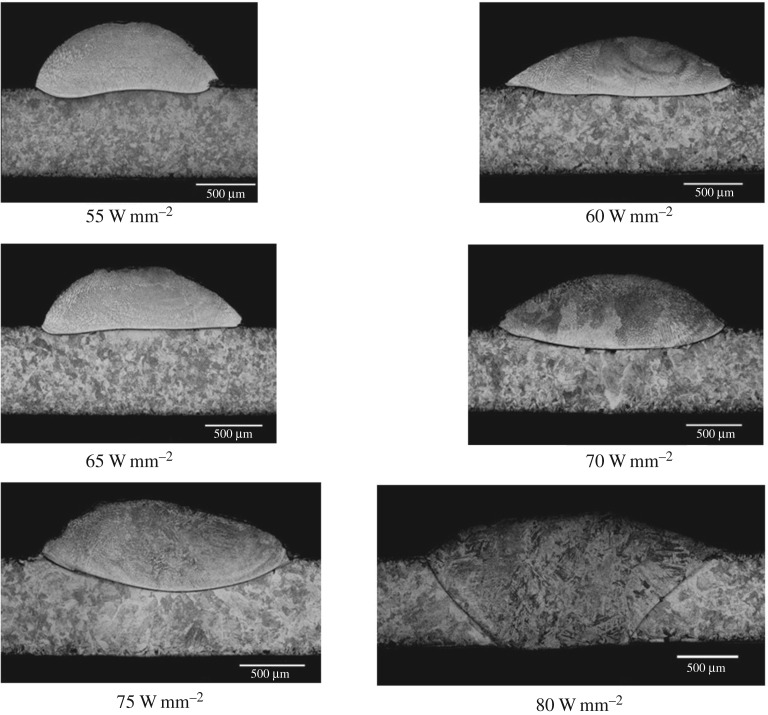
BF optical micrographs of clad tracks created with a 2** **mm square pedestal beam on pre-placed 2 × 0.3** **mm flat wire at various power densities.

Figure 7 shows the same for clad tracks created with flat wire and a pedestal laser beam. The power density range was set according to the same criteria as the round wire.

From figure 6 and 7 two observations can be made. Firstly, at the lowest power densities tested, the flat wire formed a melt pool, whereas the round wire adhered to the substrate but did not form a pool.

**Figure 8.**
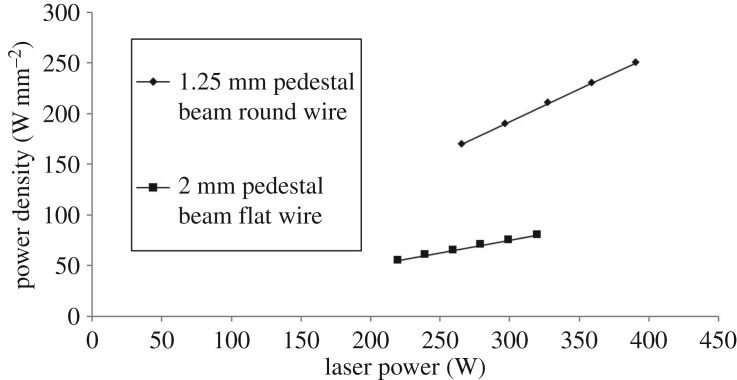
Power versus power density for 1.25** **mm square pedestal on round wire and 2** **mm square pedestal beam type on flat wire.

The relationship between power and power density for the flat wire and round wire is shown in figure 8. The use of a pedestal beam with flat wire reduces the required power density for melt pool formation compared with round wire by 68%. The large reduction in power density is largely due to the increased size of the beam, from a cross-sectional area of 1.56 mm^2^ to 4 mm^2^. This means that adequate heat input is achieved so as to melt the wire, but avoids the excessive input of heat at any one point. The reduction in power input can be accounted for by the difference in volume between the two wire types. Because the flattening process stretches the wire in two directions, both in width and in length, the cross-sectional area is reduced from 0.79 mm^2^ to 0.6 mm^2^. Because both flat and round wire were melted for the same 35 mm long track and the wire was stationary, the cross-sectional area reduction translates directly into a wire volume reduction of 24%. This actually means that slightly more energy is required per unit volume for flat wire, as opposed to round wire.

**Figure 9.**
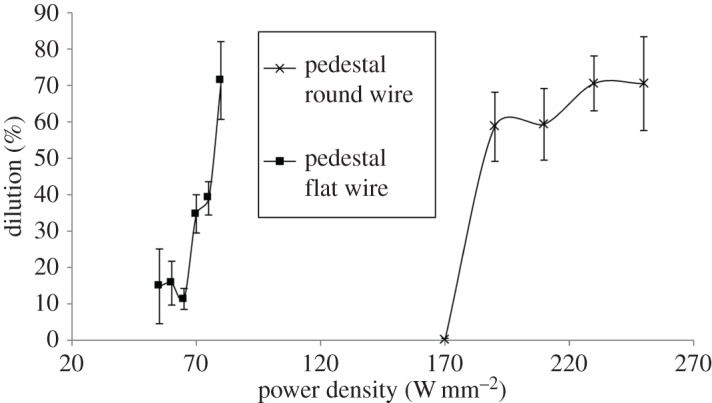
Graph of power density versus dilution for round wire and flat wire with a pedestal beam. Crosses are round wire and circles are flat wire.

In figure 9, dilution is plotted against power density for both wire types, with error bars set at 1 s.d. with five results per data point. Viewed in context with figure 8, this shows a more gentle response to increased heat input on the part of the flat wire by comparison to the flat wire.

For round wire, an increase in power density at the lowest power levels from 170 to 190 W mm^−2^ causes an increase in dilution of 58.5%. When the wire is flattened, a similar increase at low power levels from 55 to 75 W mm^−2^ gives a dilution increase of approximately 24%, less than half that of round wire, demonstrating the more gentle response of flat wire. At higher power densities, round wire appears to show more a gentle dilution response, but cross-referencing figure 9 with figure 6 shows that this is due to the fact that at high power levels the melt pool has penetrated to the far side of the substrate, impeding further dilution. When power density is converted back to power, flat wire has a range of 40 W over which the clad dilution can be controlled. For round wire, this range is approximately 10 W.

## 4. Nd:YAG laser wire-fed deposition

Nd:YAG laser cladding was carried out to create single tracks, comparing round and flat wire. Subsequent to this, flat wire was used to create multilayer clad tracks.

The two wire types required different feed rates, with a range either side at which the process was found to still be possible. If the feed rate was too low, the wire was melted before it reached the melt pool and a series of large droplets were created. If the feed rate was too high, the wire was not melted quickly enough and stubbed through the melt pool and out the other side. This is in line with results found by Abioye *et al.* [4]. The required feed rates and limits were

— round wire: 1.85 mm s^−1^ (±0.15 mm s^−1^)
— flat wire: 3.10 mm s^−1^ (±0.2 mm s^−1^).

These feed rates resulted in volume deposition rates of 1.45 mm^3^ s^−1^ for the round wire and 1.77 mm^3^ s^−1^ for the flat wire. Owing to the differences in cross-sectional area between the wire types, the difference in volume deposition rate between the two wire types was not as dramatic as the difference in feed rate would suggest, but the flat wire still showed the capability to deposit material at a 22% greater rate than round wire.

**Figure 10.**
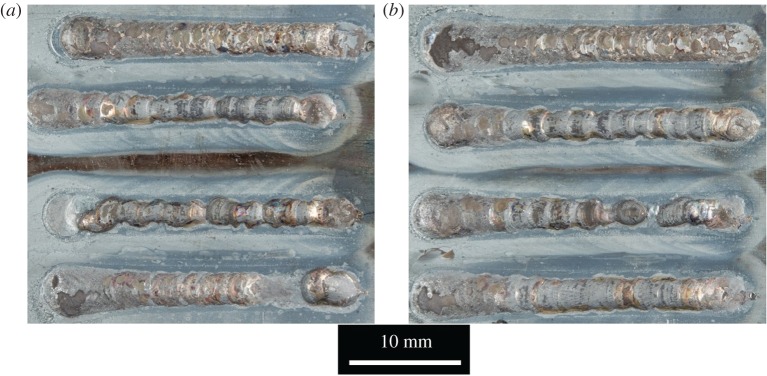
Single tracks for round wire showing (*a*) test array 1 and (*b*) test array 2.

**Figure 11.**
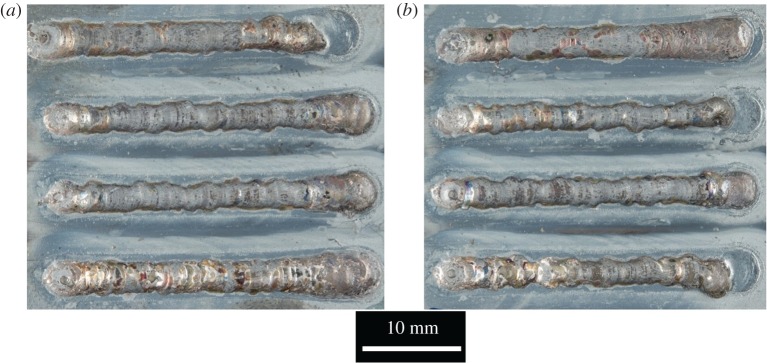
Single tracks for flat wire (*a*) test array 1 and (*b*) test array 2.

Figure 10 shows top-surface images of the two round wire arrays and figure 11 shows the equivalent results for flat wire. Both sets of tracks were created using the conditions stated in table 4, differing only in the cross-sectional shape of the wire.

A greater level of consistency in quality was found in the flat wire tracks than in the round wire tracks. The round wire tracks showed evidence of drop formation in the centre tracks for both arrays, while the flat wire tracks which maintained a continuous track in all cases. Measurements of track width and height are given in tables 5 and 6.

**Table 5.** Comparison of clad track width data for round wire versus flat wire.

	round wire	flat wire
mean track width (mm)	3.36	3.05
s.d. (mm)	0.33	0.09

**Table 6.** Comparison of clad track height above surface data for round wire versus flat wire.

	round wire	flat wire
mean track height (mm)	0.57	0.93
s.d. (mm)	0.23	0.13

Clad tracks created with flat wire showed a significantly greater level of consistency, in both height and width, as demonstrated by the differences in the standard deviation. In the case of clad track width, the standard deviation in round wire is 3.7 times that of flat wire. With track height, the difference is smaller, but still significant.

### (a) Multilayer wire-fed deposition

**Figure 12.**
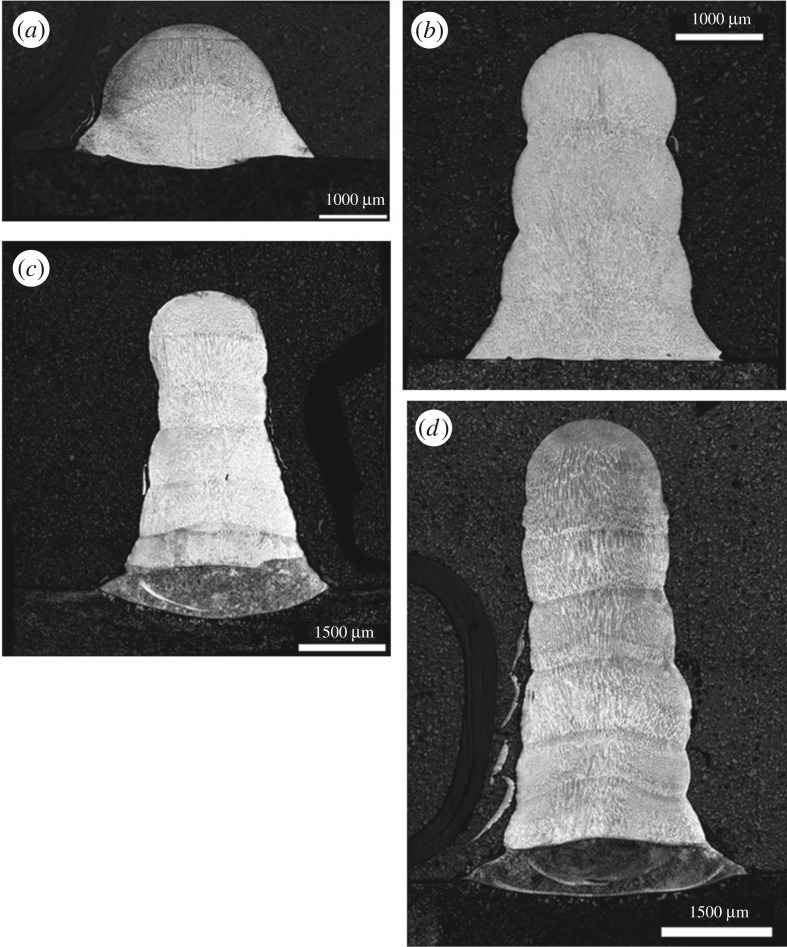
BF optical micrographs of (*a*) two-layer (*b*) four-layer (*c*) six-layer and (*d*) seven-layer clad stacks, etched with Schaftmeister's reagent.

Vertical layer stacks were created, to act as a prelude to development in a full cladding or manufacturing process (the same basic technology can be used for both). Figure 12 shows layer stacks of 2, 4, 6 and 7 layers.

In the 6 and 7 layer stacks, a significant metallurgical alteration is evident in the lowest layers. This is shown at higher magnification in figure 12.

Unlike the austenitic structure of the upper layers, the base is made up of a formation of either martensite or bainite; Vickers hardness testing with a 1 kg load was used to determine which among the latter was used. The results are given in table 7, revealing that a bainitic formation has probably occurred.

**Table 7.** Vickers hardness test results for phase change at the base of six- and seven-layer depositions.

	mean hardness (HV1)	s.d.
six-layer stack	384.6	6.3
seven-layer stack	360.6	34.8

This would normally seem out of place, because bainite does not normally occur in austenitic stainless steel. However, it is important to note that in this case, a level of mixing with the substrate has occurred. This would have the effect of locally reducing the Ni concentration in the base and, because Ni is an austenizing element, allow the decomposition of austenite that causes bainite formation to take place.

**Figure 13.**
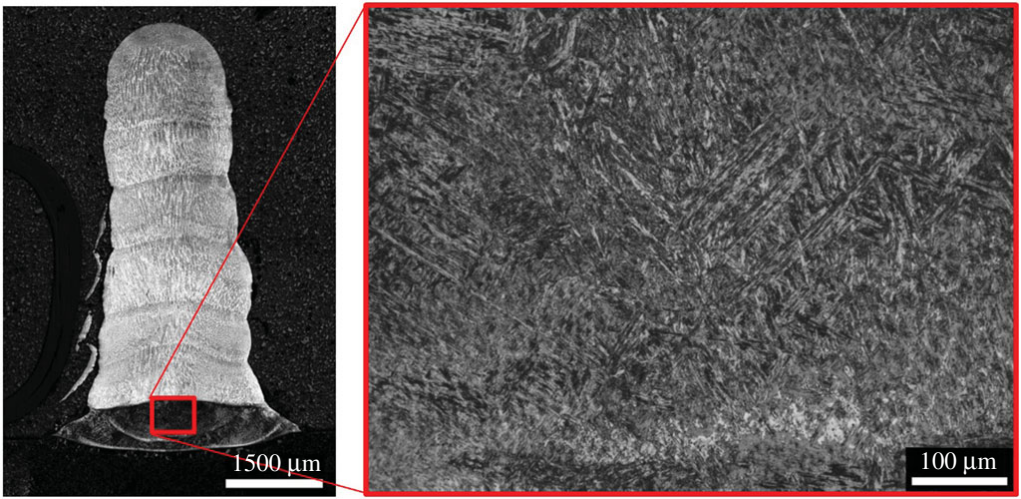
Optical micrograph showing increased magnification image of altered first layer microstructure.

**Figure 14.**
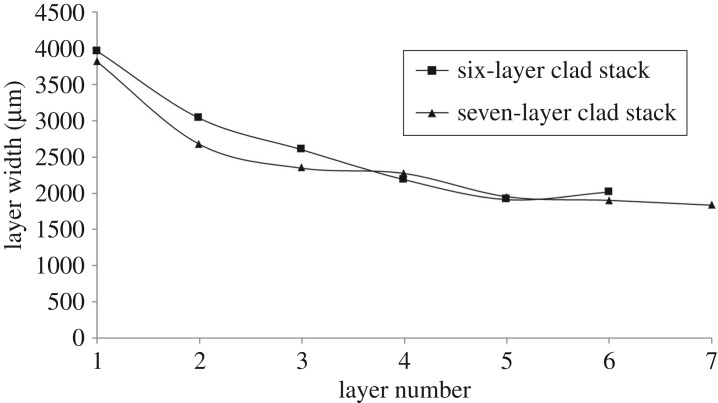
Graph correlating clad track widths with layer number.

The fact that figure 13 shows this occurring at high layer counts and not low ones suggests that this is due to repeated substrate heating by the laser beam. This repeated heating is caused by the fact that the clad stacks taper as the layer count increases. This is shown in figure 14.

**Figure 15.**
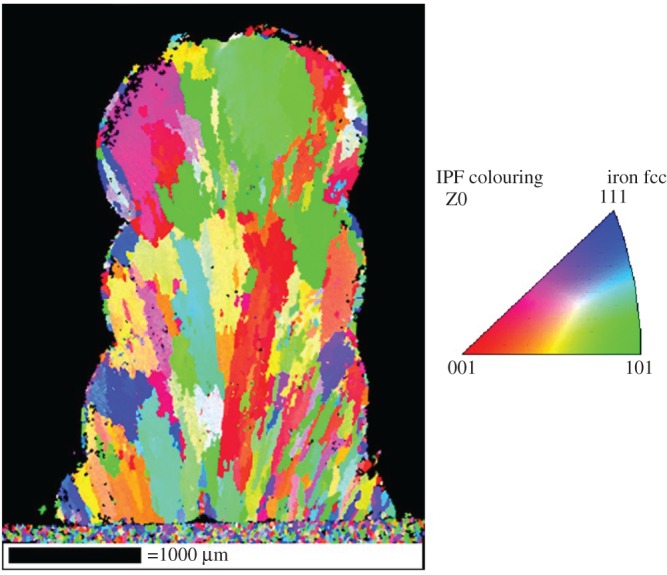
IPF maps of four-layer Nd:YAG clad track.

Grain mapping shows little regard for layer boundaries in the physical structure of the stack. Figure 15 shows an EBSD IPF map of the four layer clad stack shown in figure 13.

There is consistent anisotropic grain growth upwards from the substrate over the entire cross-section, with a wide variety of misorientation angles. Grain sizes are smaller at the bottom than at the top, proportionally to the proximity to the heat sink represented by the substrate. Lower layers cool more quickly than the upper layers, hence resulting in smaller grains. The upper layers cool more slowly and reach a higher temperature due to a reduced heat flux into the substrate, which is caused by the increased distance. The remedy for this would be to reduced laser power along with beam size as the layer count increases.

**Figure 16.**
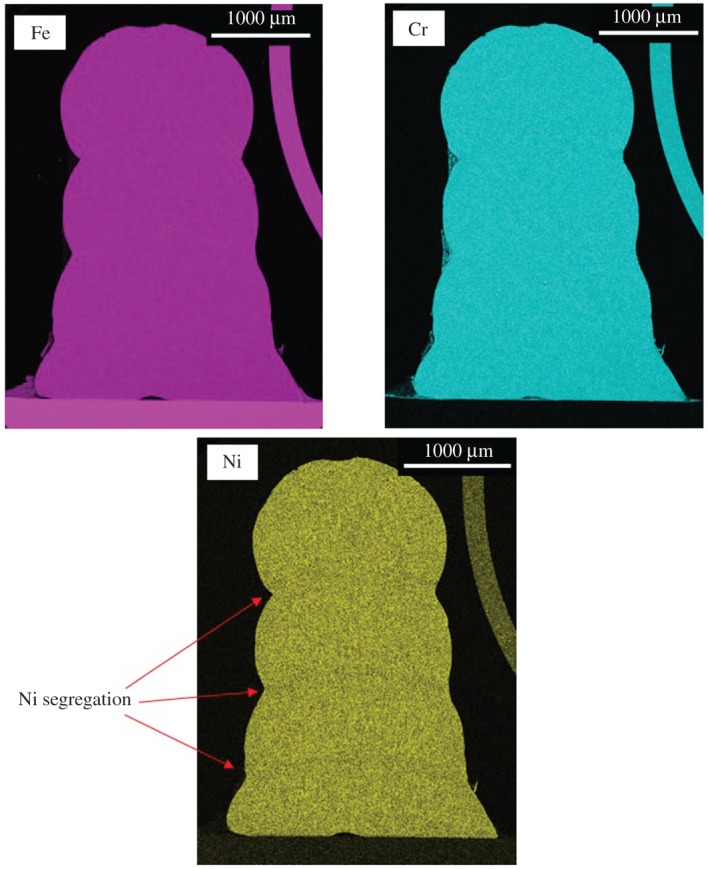
Fe, Cr and Ni EDS maps for four-layer Nd:YAG stack.

EDS maps for the four layer track are given in figure 16. Fe and Cr distribution through the stack is virtually uniform, whereas there is visible reduction in Ni concentration in the regions of the layer boundaries. This results in local Cr concentrations, leading to alterations in phase.

Measurements of element percentages reveal an overall uniformity of element distribution within the track, notwithstanding the segregation at the layer boundaries. Table 8 shows the element percentages of Fe, Cr and Ni for each layer.

**Table 8.** Layer element percentages for four-layer Nd:YAG clad stack.

	Fe (%)	Cr (%)	Ni (%)
layer 1	65.7	19.8	11.7
layer 2	64.5	19.3	11.7
layer 3	64.4	19.3	11.5
layer 4	64.8	19.3	11.6

**Figure 17.**
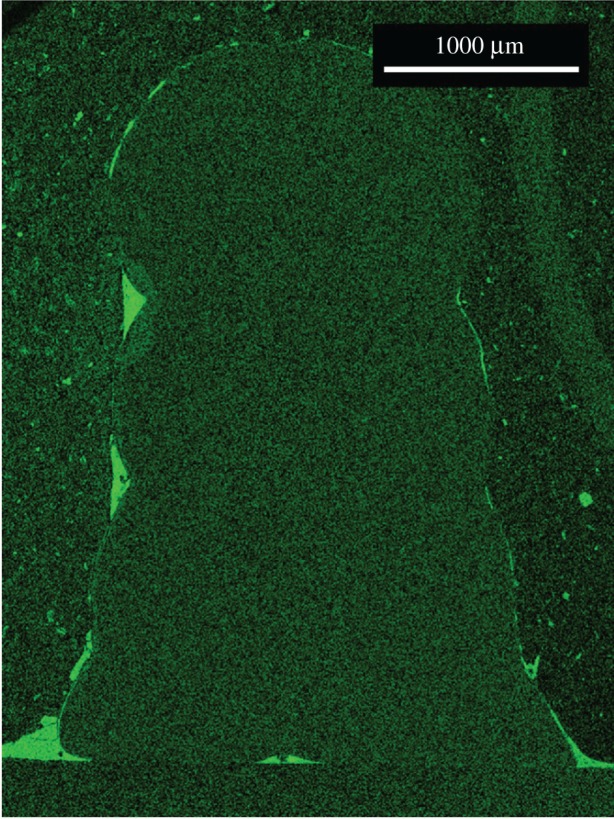
EDS oxide map of four-layer Nd:YAG clad stack.

An oxide map of the clad stack, given in figure 17, reveals that there is some O present within the structure, but that its distribution is uniform, with thin layers of oxide on the outer surface of the stack. Small oxide layers are also present between the stack and the substrate in places.

## 5. Discussion

In previous HOE work, the focus has always been on altering the shape of the beam for heat flow control and control of clad track properties of wire-based cladding. In this work, the possibility of altering wire shape in addition to beam shape has also been investigated. In addition, wire feeding was incorporated in the holographic wire deposition process for the first time. The benefits of wire shaping were therefore demonstrated in a wire-fed environment rather than being limited to the non-realistic pre-placed environment, as had been done previously.

Using shaped wire reduced the required minimum power density for melt pool formation from 170 W mm^−2^ to 55 W mm^−2^; an improvement over and above the improvement found by Goffin *et al*. when using HOEs alone on round wire. Taking the differences in beam size into account, this equates to an overall reduction in laser power from 297 W down to 220 W. Although the power density reduction was partially attributable to the reduction in wire volume, it was primarily due to the increase in beam area from 1.56 mm^2^ for round wire to 4 mm^2^ for flat wire, an expansion allowed by the increase in wire width. The greater top-surface absorption area, along with the increase in the width of the interface between the wire and substrate for the flat wire contributed to the reduced power density still allowing melting of the wire into a melt pool.

**Figure 18.**
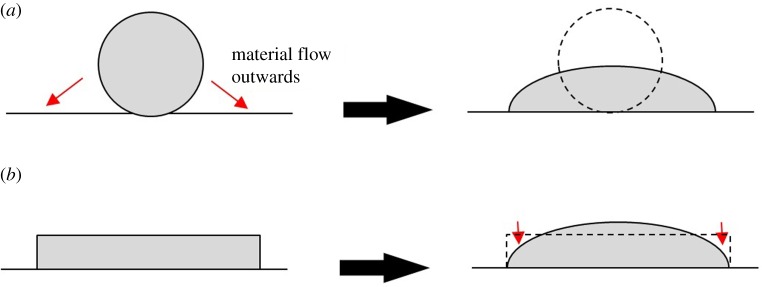
Schematic showing the level of flow required to form a melt pool for (*a*) round wire and (*b*) flat wire. Red arrows show material flow.

The differences in dilution are also partially contributed to by differences in the material flow characteristics of the two wire shapes, independent of heat input. For round wire, a melt pool forms at the wire--substrate interface, and the wire must lose its shape and flow before it melts the substrate. Until this flow is complete, bonding cannot occur at the edges. This means that the wire is diluting in the centre for longer than at the edge, leading to increased central penetration and a higher tendency to dilute with increased power density. By contrast, the flat wire is in with the substrate across its whole width so bonding occurs at all points simultaneously, giving reduced dilution once melt pool formation is complete. This is illustrated in figure 18.

The reduction of applied power density meant that flat wire tracks also showed a much reduced tendency to penetrate into the substrate, as demonstrated by the correlation of dilution with power density in figure 9. This was because the reduced power density resulted in lower temperatures in the processing zone. Uniformity of wire to substrate contact across the width of the flat wire caused it to melt and wet the substrate easily, without requiring additional time and heat for the molten wire to flow into place.

The advantages of shaped wire were found to carry over into a fed application, with the greater top-surface absorption area allowing easier melting of the wire. In wire feeding, heat transfer is via a different mechanism to pre-placed wire. It is necessary to create a substrate melt pool first, and then feed the wire into it to create the clad track. However, once the initial melt pool was achieved, it was found that flat wire gave a 22% increase in mass deposition rate versus round wire, as well as demonstrating reduced sensitivity to alterations in processing parameters. This last point is vital, because one of the primary claims found in literature regarding laser wire deposition was the inadequacy of its processing sensitivity window, which led to the general consensus of its unsuitability for industrial application and the favouring of powder-based processes. This research demonstrates the ability of flattened wire to alleviate this disadvantage.

The feeding process still remains in need of further characterization. It is probable that the higher feed rate of the flat wire was due not only to increased laser absorption but also increased heat absorption by the wire from the surrounding melt pool, giving easier melting and therefore allowing the increased wire feed rate. Initial feed rate limits were identified and provide a solid basis for further development and characterization of the wire-fed process and the benefits that wire shaping can have. Expansion of this work will be presented in future publications.

Layer stacking experiments demonstrated high-quality bonding between individual layers, while metallurgical analysis revealed that the grain structure was independent of the layer boundary position in the stack. In addition, EBSD revealed the capability to generate directional grains that followed the direction of layer growth. This shows potential for the creation of larger structures with strength-optimized metallurgy. Further metallurgical analysis with EDS showed uniformity of chemistry within the multilayer structure. As the relative proportions of Ni and Cr affect the grain structure, the uniformity of distribution of these elements within the overall lattice is vital to keep consistent material properties. The exception to this was the existence of Ni microsegregation at the layer boundaries. This means that at layer boundaries there will be an increased tendency to form ferritic rather than austenitic grains. An EDS oxide map showed the effects of oxide formation on the multilayer stack. Oxide layers were found around the outer edges of the stack. No oxide layers were present internally, having been broken up during interlayer bonding, with the O distributed through the volume of the stack. This shows that the formation of surface oxide is sufficiently limited so as to provide no impediment to interlayer bonding, meaning that full fusion occurs between layers with no weak points.

The combination of optical and SEM microscopy indicates that layer boundary properties are largely a function of chemistry rather than physical crystals. The EBSD shows that grain structure and layer boundaries are unrelated to each other, with the EDS results showing that microsegregation at the solid–liquid interface causes alterations in the chemistry at layer boundaries.

Analysis of the layer widths in figure 14 showed tapers in the multilayer stacks. This tapering is partially responsible for the repeated heating of the lower layers, causing the phase change discussed in figure 12. A number of heating mechanisms are present. Firstly, thermal inertia means that the lower layers are maintained at a high temperature for extended periods of time after their initial deposition. Two further mechanisms are then responsible for repeated heating. Firstly, the tapered shape of the stack means that the lower layers are exposed to the outer edges of the beam and repeatedly heated directly. Secondly, the heat from succeeding melt pools conducts down into the lower layers. This combination of effects results in bainite formation in the base of the stacks after a sufficient number of passes are reached. However, this would be expected to reduce as the layer count further increases. Direct heating will steadily reduce as the beam goes further and further out of focus with respect to the lower layers and at higher layer counts the distance from the top to the base of the stack is increased, reducing the effect of conduction.

With regard to the physical handling of shaped wire, the majority of the handling challenges were dependent on the wire quality. The shaping process described in §2 was developed in order to produce long lengths of flattened wire that were as straight as possible; a quality required in order to allow it to pass through the feeding system without jamming. Jams were generally caused by a kink or slight curvature in the flat wire getting caught on a feeding tube, and exacerbated by the fact that the rectangular section of the flat wire made it extremely flexible in its narrow axis, which caused it to bend further out of shape and then impede the whole feeding process. However, this narrow axis flexibility also provided advantages because it meant that the feeding nozzle assembly overall could be adjusted into more positions more easily. The greatest improvements in flat wire feeding were achieved by improvements to the shaping process. Once this was achieved, only minor modifications were required to the feed system itself. The most prominent of these was the addition of a slotted tip to the feeding nozzle in order to hold the wire at the correct orientation relative to the substrate, along with enlarged tubes to allow for the larger width of the flat wire.

## 6. Conclusion

From this work, the following conclusions have been drawn:

— Pre-placed flat wire adheres to the substrate and forms a melt pool at lower power densities (55 W mm^−2^ for flat wire versus 170 W mm^−2^ for round wire) than round wire due to greater upper and lower surface areas, reduced volume and reduced material flow. The greater upper surface area in flat wire improves absorption.
— The cross-sectional shape of pre-placed flat wire is better suited to substrate wetting than round wire, primarily due to the fact that the flat wire--substrate interface extends across the whole wire width whereas the round wire contacts the substrate only in the centre. This means that material flow is greatly reduced when the flat wire forms a melt pool compared with when the round wire forms a melt pool. A melt pool is therefore formed more easily.
— The lower power density required for melt pool creation in pre-placed flat wire means that it has a greatly reduced tendency to dilute with increases in laser power, as shown in figure 8. Flat wire therefore has a greater range of power levels over which a low-dilution track can be created.
— When flat wire is applied to wire feeding, it allows a 22% increase in the deposition rate, under identical power levels. This shows that the reduced power density requirements of pre-placed flat wire carries over into wire feeding; in the form of increased deposition for a given energy input, rather than decreased energy input for a given deposition rate.
— The use of flat wire reduces the sensitivity of the deposition process to fluctuations in processing conditions. This was demonstrated by altering the heat transfer characteristics of the substrate and then measuring the variability of the deposited tracks. Variability in flat wire tracks was reduced by a factor of 3.7 compared with round wire tracks.
— Flat wire deposition is effective in creating multi-layered stacks, the precursor to the creation of functional geometries. This process is capable of creating multiple-layer depositions where the metallurgical structure disregards layer boundaries, creating chemically homogeneous structures with uniformly distributed alloying elements and directionally controlled grain growth.

## Data accessibility

Optical micrographs and SEM data can be accessed at the Loughborough University data repository at https://dx.doi.org/10.17028/rd.lboro.4250015 [18].
